# Human Breast Cancer Cell Lines Differentially Modulate Signaling from Distant Microenvironments, Which Reflects Their Metastatic Potential

**DOI:** 10.3390/cancers16040796

**Published:** 2024-02-15

**Authors:** Ramon Ocadiz-Ruiz, Joseph T. Decker, Kate Griffin, Zoey M. Tan, Nishant K. Domala, Jacqueline S. Jeruss, Lonnie D. Shea

**Affiliations:** 1 Department of Biomedical Engineering, University of Michigan, Ann Arbor, MI 48109, USA; jocadizr@umich.edu (R.O.-R.);; 2 Department of Cariology, Restorative Sciences, and Endodontics, University of Michigan, Ann Arbor, MI 48109, USA; 3 Department of Surgery, University of Michigan, Ann Arbor, MI 48109, USA; 4 Department of Chemical Engineering, University of Michigan, Ann Arbor, MI 48109, USA

**Keywords:** metastasis, breast cancer, scaffold, implant, microenvironment, EMT

## Abstract

**Simple Summary:**

Metastasis is the stage at which the prognosis substantially decreases for many types of cancer. The ability of cancer cells to disseminate to distant organs, such as the lung, in the body is dependent on the conditioning of these distant sites by the primary tumor. Herein, we investigated the characteristics of the lung, a common site of metastasis, for human breast cancer cell lines that are either metastatic or non-metastatic. The characteristics of the lung were significantly altered by both metastatic and non-metastatic cancer cells, though the non-metastatic cells had distinct effects relative to the metastatic cells. We also investigated the use of a synthetic niche as a surrogate for the lung, which recapitulated the effects of the metastatic and non-metastatic cells. The analysis of the synthetic niche may provide an opportunity to identify disease aggressiveness without the risks associated with the biopsy of vital organs.

**Abstract:**

Metastasis is the stage at which the prognosis substantially decreases for many types of cancer. The ability of tumor cells to metastasize is dependent upon the characteristics of the tumor cells, and the conditioning of distant tissues that support colonization by metastatic cells. In this report, we investigated the systemic alterations in distant tissues caused by multiple human breast cancer cell lines and the impact of these alterations on the tumor cell phenotype. We observed that the niche within the lung, a common metastatic site, was significantly altered by MDA-MB-231, MCF7, and T47 tumors, and that the lung microenvironment stimulated, to differing extents, an epithelial-to-mesenchymal transition (EMT), reducing proliferation, increasing transendothelial migration and senescence, with no significant impact on cell death. We also investigated the ability of an implantable scaffold, which supports the formation of a distant tissue, to serve as a surrogate for the lung to identify systemic alterations. The scaffolds are conditioned by the primary tumor similarly to the lung for each tumor type, evidenced by promoting a pro-EMT profile. Collectively, we demonstrate that metastatic and non-metastatic breast cancers condition distant tissues, with distinct effects on tumor cell responses, and that a surrogate tissue can distinguish the metastatic potential of human breast cancer cell lines in an accessible site that avoids biopsy of a vital organ.

## 1. Introduction

Breast cancer has surpassed lung cancer as the most diagnosed type of cancer, with approximately 2.3 million new cases globally [1]. Metastatic spread of the disease occurs in approximately 25% of cases, contributing to more than 43,000 deaths per year in the USA [2]. Metastatic disease is associated with the dissemination of tumor cells from the primary neoplasm to secondary sites, predominately to bones, lungs, liver, lymph nodes, and brain, drastically reducing patient survival [3]. One of the reasons underlying the impact of metastatic disease is that detection occurs after a patient self-reports symptoms, and the identification of biomarkers for early detection of metastatic disease could allow treatment while the disease burden and tumor cell heterogeneity are low. However, biomarkers for early detection have been challenging to develop as metastasis can occur over varying time scales, ranging from months to decades, depending on the biology of the tumor and treatment [3,4].

Metastasis reflects a combination of both tumor-cell-intrinsic and -extrinsic elements that regulate metastatic seeding, development, and progression, as initially described with Paget’s “seed and soil” hypothesis. Intercellular signaling between the tumor and host cells within the metastatic niche drives recruitment, colonization, and ultimately progression of disease [5]. Among the tumor-cell-intrinsic elements that regulate distant disease are mutation accumulation; epigenetic plasticity driven by chromosomal instability; and phenotypic states like epithelial–mesenchymal transition (EMT), which promote tumor cell migration, cell senescence, and dormancy [6,7,8,9]. The extrinsic factors reflect the composition of the extracellular environment, and can include immune cell composition, and the release of immunomodulatory cytokines, chemoattractants, and growth factors that can lead to a microenvironment favorable for tumor cell colonization. The niche within the distant tissue is influenced by the characteristics of the primary tumor, which can dysregulate immune processes systemically [10,11]. Hence, the characteristics of these distant tissues could encode valuable information about their potential to support metastatic progression, with this information providing opportunities to detect disease or identify therapeutic targets.

In this report, we analyzed the systemic alterations in distant tissues caused by the primary tumor for a selection of human breast cancer types with a range of metastatic potential. Human breast cancer cell lines were inoculated into the mammary fat pad. Primary tumor, bone marrow, and lungs were collected and employed to generate conditioned media representative of the primary tumor and metastatic sites. In vitro assays, including cell proliferation, apoptosis, transendothelial migration (TEM), senescence, and stemness, were evaluated using the conditioned media in contact with tumor cells. Signaling pathways stimulated by this communication were analyzed using a TRanscriptional Activity Cell aRray (TRACER) [12,13], which identified that all primary tumors modified the environment in distant tissues, altering responses such as tumor cell proliferation, migration, senescence, and stemness. Distant tissues collected from mice with the most aggressive primary tumors demonstrated the greatest impact on tumor cell responses. The observation that all human tumor cell lines influence the microenvironment in distant tissues led to an investigation of whether this conditioning could also be identified using a synthetic metastatic niche. We have developed a porous biomaterial implant, which supports vascularization and infiltration of immune and tumor cells, thus functioning as a synthetic metastatic niche [14]. The synthetic niche recapitulated the conditioning by the primary tumor of endogenous tissues. The synthetic metastatic niche thus provides an opportunity to assess the metastatic potential of a primary tumor without invasive biopsy of a vital organ.

## 2. Methods

### 2.1. Cell Culture

Human breast cancer cell lines MDA-MB-231 and MCF-7 (ATCC; Manassas, VA, USA) were cultured in Advanced RPMI 1640 medium (Gibco; Waltham, MA, USA) supplemented with 10% fetal bovine serum (FBS) in a humidified 5% CO_2_ incubator at 37 °C. Media were changed every 3 days, once ~80% confluent, cells were harvested with TrypLE Express (Life Technologies; Carlsbad, CA, USA) solution and counted using Trypan blue stain (Sigma Aldrich; St. Louis, MO, USA) and a Cell Countess automated hemocytometer (Life Technologies). Cells were routinely tested for Mycoplasma contamination using the PCR-based Universal Mycoplasma Detection Kit (ATCC). Cell lines were authenticated by short tandem repeat DNA analysis and compared to the ATCC STR profile database (DDC Medical; Manassas, VA, USA).

### 2.2. PCL Scaffold Fabrication

Microporous poly(ε-caprolactone) (PCL) scaffolds were fabricated by salt leaching technique as previously described [14,15,16,17]. PCL microspheres were mixed with sodium chloride particles (250–425 μm) at a 1:30 (*w*/*w*) ratio in a mixer (ROSS) for 30 min at 85 °C. Subsequently, 6.2 g of polymer/salt mixture was placed in a 40 mm diameter steel die and pressed at 1500 psi for 45 s in bench top manual press (Carver; Wabash, IN, USA) to form a polymer/salt disc (2 mm in height). Scaffold leaching was performed with distilled water overnight, and polymer discs were placed on dry ice and cut at 5 mm diameter (Miltex; York, PA, USA). Scaffolds were disinfected with 70% ethanol and stored at −80 °C until implantation. Scaffolds are 90% porous; the homogeneous porous distribution allows cell infiltration of about 75%.

### 2.3. Subcutaneous Scaffold Implantation

Animal studies were performed in accordance with the University of Michigan Institutional Animal Care and Use guidelines and protocols. Poly(ε-caprolactone) (PCL) scaffolds were implanted into the dorsal subcutaneous space of female NOD/SCID-IL2R γ−/− (NSG, Jackson Laboratory; Bar Harbor, ME, USA) mice (7–10 weeks old). Mice were implanted with two 5 mm scaffolds, and wounds were closed using surgical stainless-steel clips (ThermoFisher; Waltham, MA, USA). Carprofen was administered postoperatively every 24 h for 48 h. *n* = 5 mice per condition.

### 2.4. Estradiol Supplementation

As HER2-positive human cancer cell lines require human estrogen to form tumors, mice were subcutaneously implanted in the lateral side of the neck with a pellet of 0.18 mg of 17-beta-estradiol using a stainless-steel trocar (Innovative Research of America; Sarasota, FL, USA). Incision was closed with tissue adhesive and surgical stainless-steel clips (ThermoFisher).

### 2.5. Tumor Cell Inoculation

Human breast cancer cells MDA-MB-231 or MCF-7 (2 × 10^6^) were resuspended in growth factor free Matrigel (Corning; Corning, NY, USA) and inoculated into the right mammary fat pad of female NOD/SCID-IL2R γ−/− (NSG) mice. The endpoint for conditioned media preparation was 30 days after tumor cell inoculation. *n* = 5 mice per condition.

### 2.6. Conditioned Media Preparation

Tissues derived from tumor-free or tumor-bearing mice after 30 days of TCs inoculation and when the primary tumor reached a size between 0.5 and 1 cm [15,18]. Tissues were processed into single-cell suspensions. Tissues were minced with scalpel blades, enzymatically digested with Liberase TM (Roche; Basel, Switzerland), and passed through a 100 µm cell strainer. Cell suspensions underwent erythrocyte lysis using ACK lysis buffer (Gibco) for 5 min. Cells were washed with PBS and resuspended in FBS-free media (phenol red-free RMPI-1640 medium) at a concentration of ~2 × 10^6^ cells/mL and incubated at 37 °C for 24 h. After incubation, supernatant was collected by centrifugation and stored at −80 °C. Conditioned media total protein concentration was determined by BCA protein assay kit (ThermoFisher) and adjusted to a final concentration of 0.3 mg/mL for in vitro studies.

### 2.7. Cell Proliferation and Apoptosis

TC proliferation was evaluated using the Click-iT EdU Cell Proliferation Kit (ThermoFishe; Waltham, MA, USA). EdU solution was added into media of TCs incubated with conditioned media from either tumor-free or tumor-bearing tissues, after 8 h of incubation cells were fixed and permeabilized for later EdU detection following manufacturer indications. Images were taken by a fluorescence microscope (Zeiss; Jena, Germany). TC cell death by apoptosis was evaluated using the APO-BrdU TUNEL Assay Kit (Invitrogen; Waltham, MA, USA). BrdU solution was added into media of TCs with conditioned media from either tumor-free or tumor-bearing tissues, 24 h later cells were fixed and processed according to manufacture guidelines. Images were taken by a fluorescence microscope (Zeiss; Jena, Germany). Analysis and cell quantification were performed using the cell image analysis software CellProfiler version 4.2.6 [19,20]. *n* = 4 with triplicates per condition.

### 2.8. Senescence

Cellular senescence was analyzed by quantitative RT-PCR (qRT-PCR) to measure and compare the gene expression of the cell senescence markers CDKN1A (Hs00355782_m, Taqman, ThermoFisher), CDKN2A (Hs00923894_m1, Taqman, ThermoFisher), and CDKN2B (Hs00793225_m1, Taqman, ThermoFisher) of TCs incubated with conditioned media from either tumor-free or tumor-bearing tissues. Transcriptional levels were normalized with internal controls GAPDH (Hs02786624_g1, Taqman, ThermoFisher) and 18S (Hs99999901_s1, Taqman, ThermoFisher). A minimum of four biological replicates per assay were analyzed. *n* = 4 with triplicates per condition.

### 2.9. Stemness

TC stemness activity was determined by mammosphere assays. TCs were plated in ultra-low attachment plates (Corning) in conditioned media from either tumor-free or tumor-bearing tissues. Tumor spheres (>50 µm) were counted, and control groups were included with TCs incubated with complete Advanced RPMI 1640 medium (Gibco) supplemented with 10% fetal bovine serum (FBS). A minimum of four biological replicates per assay were analyzed. *n* = 4 with triplicates per condition.

### 2.10. Transendothelial Migration

Conditioned media derived from lung, and primary tumors of mice inoculated with MDA-MB-231 tumor cells, were used as a chemoattractant agent at the bottom of the transwell chamber where MDA-MB-231 cells were placed over a monolayer of activated Human Umbilical Vein Endothelial cells (HUVECs) in the upper membrane of the transwell. Two days after TC incubation, transmigrated cancer cells were stained with DAPI and fixed with 4% PFA. Images were taken by a fluorescence microscope (Zeiss; Jena, Germany). Analysis and cell quantification were performed using the cell image analysis software CellProfiler version 4.2.6 [19,20]. *n* = 4 with triplicates per condition.

### 2.11. Flow Cytometry

Flow cytometry of the lung and scaffolds was performed following the previously published protocol [21]. In brief, scaffolds and lung single cells were isolated by enzymatic digestion with 1 mg/mL collagenase A (Roche; Indianapolis, IN, USA) and 20 U/mL DNase I (Sigma, St. Louis, MO, USA) in RPMI 1640 containing 10% fetal calf serum (FCS). Tissues were further dispersed through an 18-gauge needle (10 mL syringe), RBCs were lysed, and samples were filtered through 100 μm nylon mesh twice. Cells were resuspended in PBS, and live cells were identified using LIVE/DEAD Fixable Yellow Dead Cell Stain kit (Thermo Fisher Scientific), then washed and resuspended in PBS with 1% FCS, and Fc receptors were blocked with purified anti-CD16/32 (clone 93; BioLegend; San Diego, CA, USA). Surface markers were identified using Abs (clones) against the following antigens, all from BioLegend: anti-Gr-1 (RB6- 8C5), CD64 (X54-5/7.1), sinCD11b (M1/70), c-Kit (2B8), CD11c (N418), MHCII (M5/114.15.2), SiglecF (S17007L), F4/80 (BM8), Ly6C (HK1.4). For Neutrophils: SSChigh CD11b+ SiglecF- GR-1+. Antigen-presenting cells (APC) DC CD11b+CD11c+: CD64-CD11b+CD11c+MHCII+, Macrophages: CD11b+CD64+F4/80+SiglecF-. Ly6Chigh monocyte: CD45+CD11b+SiglecF- Ly6Chigh. We provided a representative cell flow cytometry gating strategy (Supplementary Figure S2). Data were collected using a NovoCyte flow cytometer (ACEA Bioscience, Inc. San Diego, CA, USA). Data analysis was performed using FlowJo software version 10.10 (Tree Star, Ashland, OR, USA). Data were analyzed by Prism 8 (GraphPad Software). Data presented are mean values ± S.E.M. Comparison of two groups was performed with an unpaired, two-tailed Student *t*-test. Comparisons of three or more groups were analyzed by ANOVA with a Tukey posttest. A *p*-value less than 0.05 was considered significant. *n* = 3 with triplicates per condition.

### 2.12. TRanscriptional Activity Cell aRray (TRACER)

The tumor cells’ transcriptional activity profile was studied using the dynamic TRanscriptional Activity CELL aRray (TRACER) [12,22]. The transcription factor (TrF) reporters delivered in self-inactivating lentivirus consist of TrF response elements cloned upstream of a minimal thymidine kinase promoter that expresses the firefly luciferase protein (Fluc). The reporters’ design and construction have been described in detail previously [12,22]. MDA-MB-231 cells were transduced with lentiviral reporter constructs at a multiplicity of infection (MOI) of 80 and seeded in black 384-well plates (Greiner Bio-One) at a density of 2 × 10^3^ cells/well and cultured for a minimum of 48 h. Each well receives a single lentiviral reporter with a minimum of four technical replicates. After 48 h, media was replaced with conditioned media (Lung, PT, and scaffold) supplemented with 1 mM D-Luciferin (Promega Corporation; Madison, WI, USA) for subsequent Fluc activity measurement by bioluminescence imaging (Perkin Elmer IVIS Spectrum; Waltham, MA, USA). Bioluminescence intensity (BLI) was measured at several time points (0, 1, 2, 4, and 6 h) based on preliminary experiments using this culture system. TRACER analysis was performed by normalizing each TrF activity signal and statistical significance was determined using previously described methodology [12]. Each timepoint background signal measured in a non-transduced cell control was subtracted from each BLI measurement and then normalized to each corresponding condition’s TA control reporter to obtain the log2 fold-change in BLI. Heatmaps were generated by averaging the replicate log2 fold-change for each reporter condition and time point. Statistical analysis was performed using the limma R (4.3) package [23].

### 2.13. Statistical Analysis

Data are shown as mean ± standard error (SEM) unless otherwise noted. Significance was determined by *p*-values less than 0.05, using unpaired Student’s *t*-test for single comparisons or one-way ANOVA with Bonferroni testing for multiple comparisons. Fisher’s exact test was used to test the significance between TrF values, with *n* = 4 replicates per condition. Statistical analysis was performed using GraphPad Prism version 10.2.0.

## 3. Results

### 3.1. Innate Immune Cell Recruitment in the Endogenous Metastatic Niche of MDA-MB-231 Tumors Supports EMT

**Figure 1:**
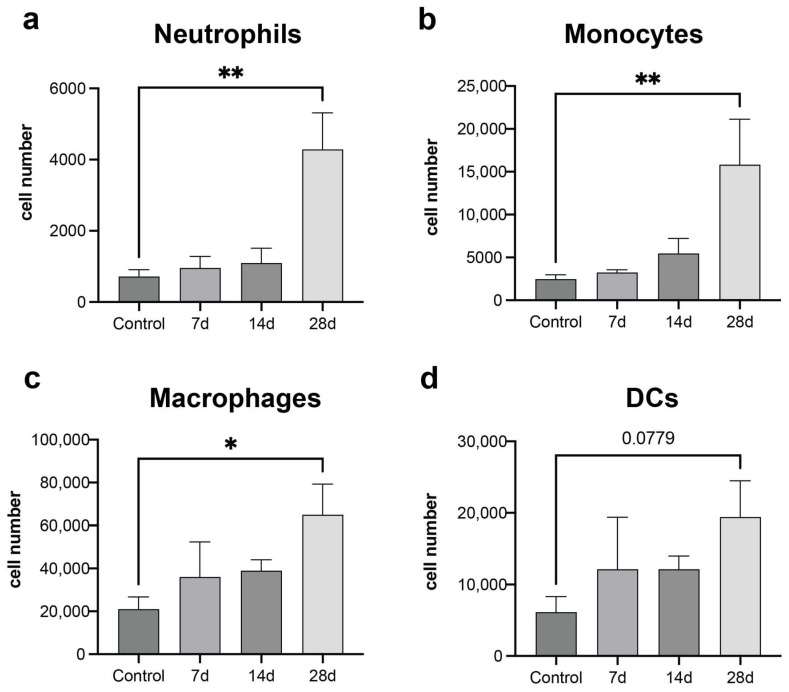
High immune cell recruitment in the natural metastatic niche of MDA-MB-231 inoculated mice. Flow cytometry characterization of the innate immune cell response in the lung of MDA-MB-231 inoculated mice: (**a**) Neutrophils: SSC^high^ CD11b+ SiglecF- GR-1+, (**b**) Monocytes: CD45+CD11b+SiglecF- Ly6C^high^, (**c**) Macrophages: CD11b+CD64+F4/80+SiglecF-, and (**d**) Dendritic cells: CD64-CD11b+CD11c+MHCII+. Comparison of two groups was performed with an unpaired, two-tailed Student *t*-test. Comparisons of three or more groups were analyzed by ANOVA with a Tukey posttest. * *p* < 0.05, ** *p* < 0.01.

We characterized the innate immune cell composition within the lung, the endogenous metastatic niche, across disease progression. Innate immune cells, including macrophages, dendritic cells, neutrophils, and monocytes, have been reported to play a role in tumor progression and metastasis, and their abundance at metastatic sites can be directed by the primary tumor (PT). MDA-MB-231 cells, an aggressive triple-negative breast cancer cell line, were inoculated into female NOD/SCID-IL2R γ−/− (NSG) mice, and the lung was harvested and analyzed by flow cytometry at 7-, 14-, and 28-days post-inoculation. An increased number of neutrophils (SSChigh CD11b+ SiglecF- GR-1+), monocytes (CD45+ CD11b+ SiglecF- Ly6Chigh), and macrophages (CD11b+ CD64+ F4/80+ SiglecF-) were observed in the lung at day 28 post-tumor-cell inoculation relative to the tumor-free controls (Figure 1a–c) This time point correlates with the appearance of micrometastatic lesions in the lungs. The Day 7 and 14 time points did not demonstrate significant increases, yet a trend toward gradually increasing numbers over time was observed. Dendritic cells (CD64-CD11b+CD11c+MHCII+) were not observed to significantly increase in the lung by Day 28, though a trend was observed, yet was not significant, due to the increased variability (Figure 1d). High immune cell infiltration was also observed in the lungs and scaffolds of tumor-bearing mice (Supplementary Figure S3).

**Figure 2:**
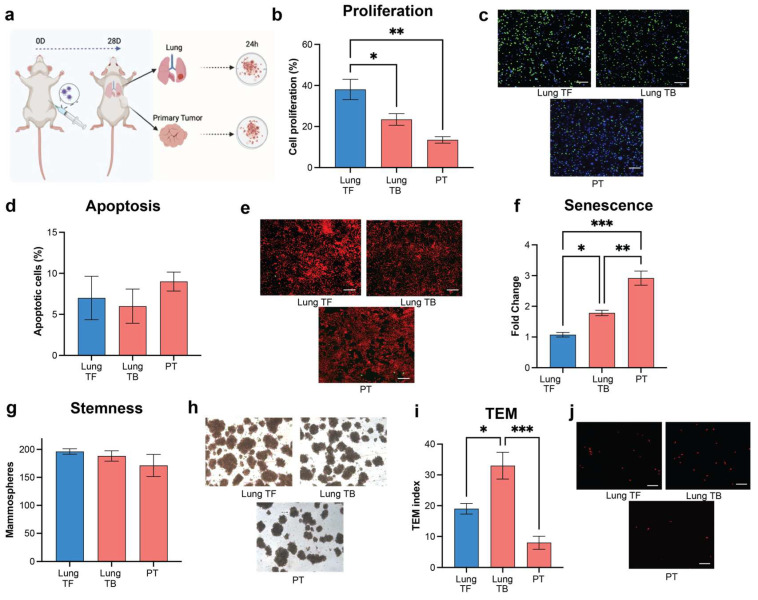
Metastatic niche microenvironment supports a tumor cell EMT phenotype. Functional analysis of healthy and tumor-induced tissue microenvironment on tumor cell phenotype: (**a**) Schematic of conditioned media production from single cell suspension of healthy lung tumor-free (TF) and tumor-bearing (TB) primary tumor and lung. Created using Biorender.com (**b**) Cell proliferation assessment of MDA-MB-231 tumor cells incubated with healthy and tumor-induced microenvironment tissues. (**c**) Representative immunofluorescent images of the cell proliferation assay, (**d**) Apoptosis assay, (**e**) Representative immunofluorescent images of TUNEL assay, (**f**) Senescence, (**g**) Stemness, (**h**) Representative images of MDA-MB-231 mammospheres, (**i**) Migration (TEM) assay, and (**j**) Representative immunofluorescent images of the Transendothelial Migration assay. Comparison of two groups was performed with an unpaired, two-tailed Student *t*-test. Comparisons of three or more groups were analyzed by ANOVA with a Tukey posttest. * *p* < 0.05, ** *p* < 0.01, *** *p* < 0.001.

We assessed the influence of a metastatic microenvironment on the cancer cell phenotype through functional analysis using an ex vivo culture. Lungs and primary tumors of mice bearing MDA-MB-231 tumors were isolated and a single-cell suspension was created to produce conditioned media (CM) (Figure 2a). Lung tissues from tumor-free animals were similarly processed as a control. Proliferation of MDA-MB-231 cells was significantly reduced by incubation with CM derived from the PT and lungs of tumor-bearing mice in comparison to CM from tumor-free mice (Figure 2b,c). Apoptosis of the tumor cells in all conditioned media was generally low. No significant changes were observed among tissues from tumor-bearing and tumor-free mice (Figure 2d,e). Interestingly, cell senescence in the MDA-MB-231 cells cultured with CM from PT and lungs of tumor-bearing animals was increased relative to CM from the lungs of tumor-free mice (Figure 2f).

Stemness induced by the microenvironment was analyzed by the ability of MDA-MB-231 cells to form spheroids. A high stemness index was maintained across all microenvironment conditions, with no significant changes between conditions (Figure 2g,h). Notably, a significant increase in trans-endothelial migration (TEM) was observed when tumor cells were exposed to the lung microenvironment from tumor-bearing mice, compared to the cells incubated with PT or with a healthy lung microenvironment (Figure 2i,j). The metastatic niche microenvironment promotes a significant increase in TEM activity compared to the PT or healthy lung microenvironment. Collectively, these studies suggest that the metastatic microenvironment influences the phenotypic responses of MDA-MB-231 cells, with notable differences between the primary tumor and lung.

### 3.2. Microenvironment from Highly Metastatic Tumors Induces EMT Signaling

**Figure 3:**
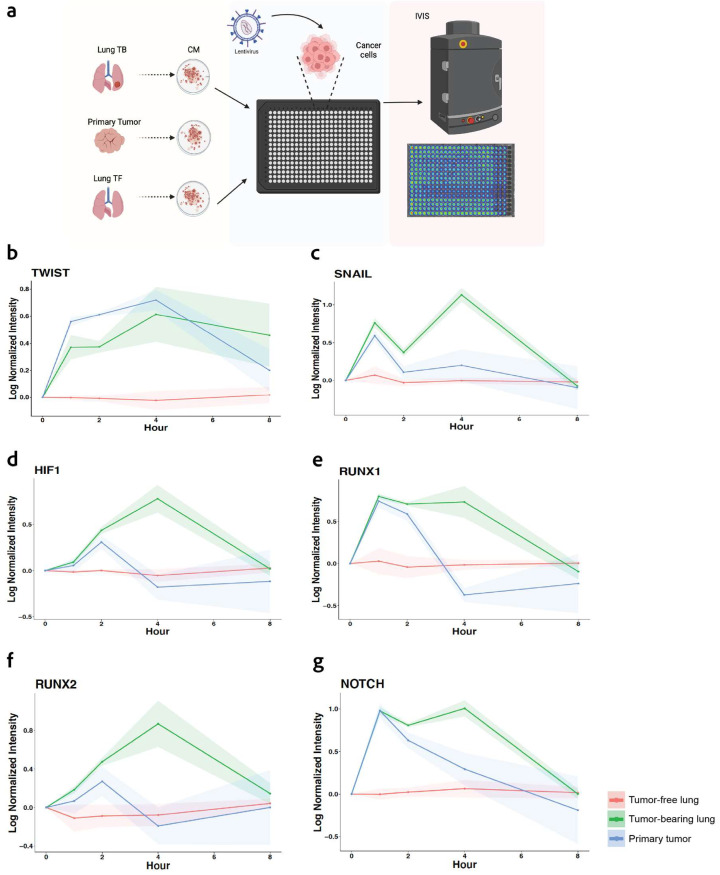
High metastatic microenvironment induces EMT-related transcription factor (TrF) activity: (**a**) Dynamic transcriptional activity of EMT signaling of MDA-MB-231 tumor cells incubated with CM from healthy and tumor-bearing tissues. Tumor cells are transduced with reporters and seeded into a well plate, with each well having a singular reporter. Condition media was added to the tumor cells as indicated. TrF activity for (**b**) TWIST1, (**c**) SNAIL, (**d**) HIF1a, (**e**) RUNX1, (**f**) RUNX2, and (**g**) NOTCH1. The shaded areas for each line indicate the standard deviation from the mean. *n* = 4 replicates per condition.

We subsequently investigated the transcriptional activity associated with the epithelial-to-mesenchymal transition (EMT) phenotype, which is a process that enables epithelial cells to acquire mesenchymal cell properties, enhancing their migratory capacity, invasiveness, and resistance to apoptosis. EMT is characterized by several cellular responses, including alterations in proliferation, senescence, stemness, and migration that were analyzed above. We used a real-time transcriptional activity assay known as TRACER (TRanscriptional Activity CELL aRray) to measure the dynamic activity of six transcription factors involved in EMT signaling specific for breast cancer (TWIST1, SNAIL, HIF1a, RUNX1, RUNX2, and NOTCH1) [12,24]. MDA-MB-231 cells were first transduced with a reporter construct and subsequently cultured with conditioned media derived from tumor-free lungs, or from lungs and PT obtained from tumor-bearing mice at day 28. Luminescence was monitored over time to quantify the transcriptional activity (Figure 3a). An increase in the EMT-associated Transcription Factor (TrF) activity was observed for the culture with tumor-bearing CM relative to the tumor-free CM (Figure 3). All the analyzed TrFs from tumor-bearing microenvironments showed an activation at early timepoints, between 1 and 2 h, relative to tumor-free conditions. Particularly, Twist1 exhibited sustained activation at early timepoints in both PT and lung microenvironment that lasted several hours (Figure 3b). In contrast, SNAIL, HIF1, RUNX1, RUNX2, and NOTCH1 displayed transient activity early in the PT group. Yet, the CM from the lungs of tumor-bearing mice presented a consistent TrF activation until the 8 h time point (Figure 3c–g). These data confirm the premise that the conditioning of the lung by the PT presents signals that lead to the persistent activation of TrFs that are associated with EMT, which aligns with a more invasive and migratory phenotype in the tumor cells.

### 3.3. Scaffolds Recapitulate the Natural Metastatic Microenvironment Effect in the TC Phenotype

Our observation that metastatic human breast cancer cell lines influence the microenvironment in distant tissues led to an investigation into whether this conditioning effect by metastatic tumors could be detected in an engineered tissue. We have previously reported the development of a microporous scaffold that becomes infiltrated with cells and vascularized upon implantation [13]. Herein, this technology was employed to evaluate the conditioning effect of human tumors on the synthetic niche relative to the endogenous tissue (lung), and to examine the impact of these recruited cells on the tumor cell phenotype using the ex vivo culture system.

**Figure 4:**
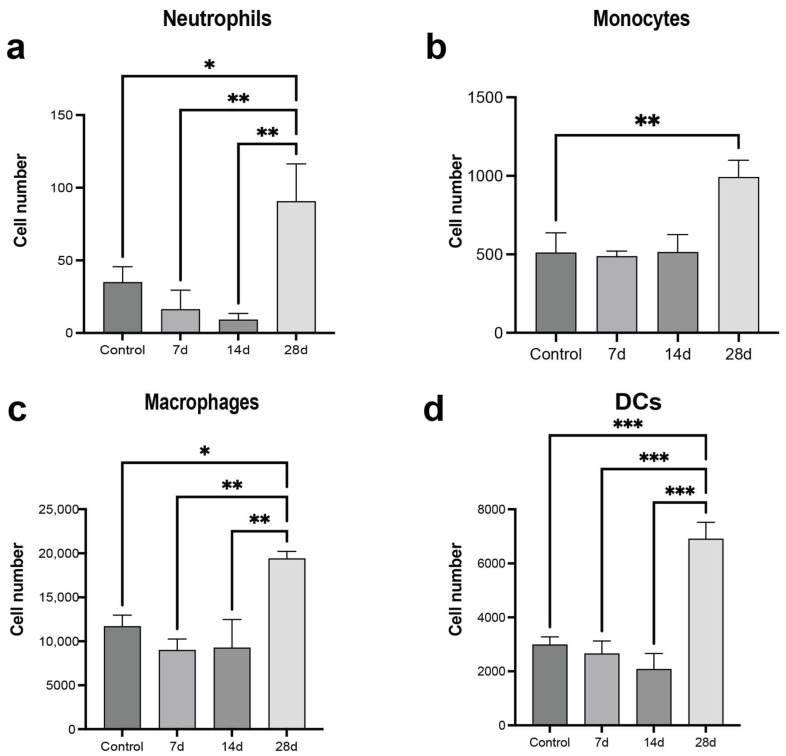
Scaffolds recapitulate the immune cell recruitment in the natural metastatic niche. Characterization of the Innate immune cell response in scaffolds implanted in healthy or tumor-bearing mice: (**a**) Neutrophils: SSC^high^ CD11b+ SiglecF- GR-1+, (**b**) Monocytes: CD45+CD11b+SiglecF- Ly6C^high^, (**c**) Macrophages: CD11b+CD64+F4/80+SiglecF-, and (**d**) Dendritic cells: CD64-CD11b+CD11c+MHCII+. Comparison of two groups was performed with an unpaired, two-tailed Student *t*-test. Comparisons of three or more groups were analyzed by ANOVA with a Tukey posttest. * *p* < 0.05, ** *p* < 0.01, *** *p* < 0.001.

**Figure 5:**
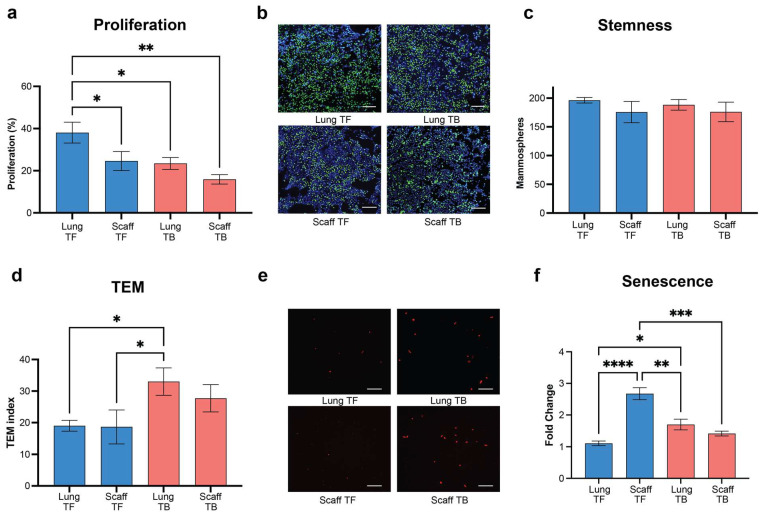
Synthetic metastatic niche emulates the endogenous metastatic effect on tumor cell phenotype. Functional analysis of healthy and tumor-induced natural and synthetic microenvironment on tumor cells: (**a**) Cell proliferation assessment of MDA-MB-231 tumor cells incubated with tumor-induced microenvironment tissues. (**b**) Representative immunofluorescent images of the cell proliferation assay (**c**) Stemness, (**d**) Migration (TEM) assay. (**e**) Representative immunofluorescent images of the Transendothelial Migration assay. (**f**) Senescence. Comparison of two groups was performed with an unpaired, two-tailed Student *t*-test. Comparisons of three or more groups were analyzed by ANOVA with a Tukey posttest. * *p* < 0.05, ** *p* < 0.01, *** *p* < 0.001, **** *p* < 0.0001.

Following TC inoculation, we analyzed the innate immune composition in the synthetic niche. An increased number of macrophages, monocytes, neutrophils, and DCs were observed at day 28 post-tumor-cell inoculation relative to tumor-free mice, consistent with the results in the lung (Figure 4a–d). The earlier time points had some differences relative to the lung. Neutrophils and macrophages had modest decreases in cell numbers at day 7 and 14 relative to the tumor-free mice. We also assessed the phenotypic responses induced by the microenvironment of the scaffold. CM was derived from lungs and scaffolds implanted in mice that had been inoculated with MDA-MB-231 cells, and cultured ex vivo with the same MDA-MB-231 cells. CM from scaffolds of tumor-bearing mice significantly reduced tumor cell proliferation compared to scaffolds from tumor-free mice. Moreover, the cell responses to the CM from the scaffold were remarkably similar to what was observed with the conditioned media from the lungs of MDA-MB-231-tumor-bearing mice (Figure 5a,b). Stemness of the TCs was high with no significant variations among CM from healthy or tumor-bearing mice (Figure 5c). TEM measured with CM from the scaffold of tumor-bearing mice was comparable to CM from the lung of tumor-bearing animals, but higher relative to CM from scaffolds of healthy mice (Figure 5d,e). Senescence was comparable for both CM from the scaffold and endogenous metastatic niche from MDA-MB-231-inoculated mice (Figure 5f). These results indicate that the scaffolds recapitulate the endogenous metastatic niche. They reproduce the cellular recruitment and signaling patterns observed in distant natural metastatic sites like the lung and influence the tumor cell phenotype.

### 3.4. Non-Metastatic Tumors Condition Distant Tissues and Support Pro-Cancer Signaling

**Figure 6:**
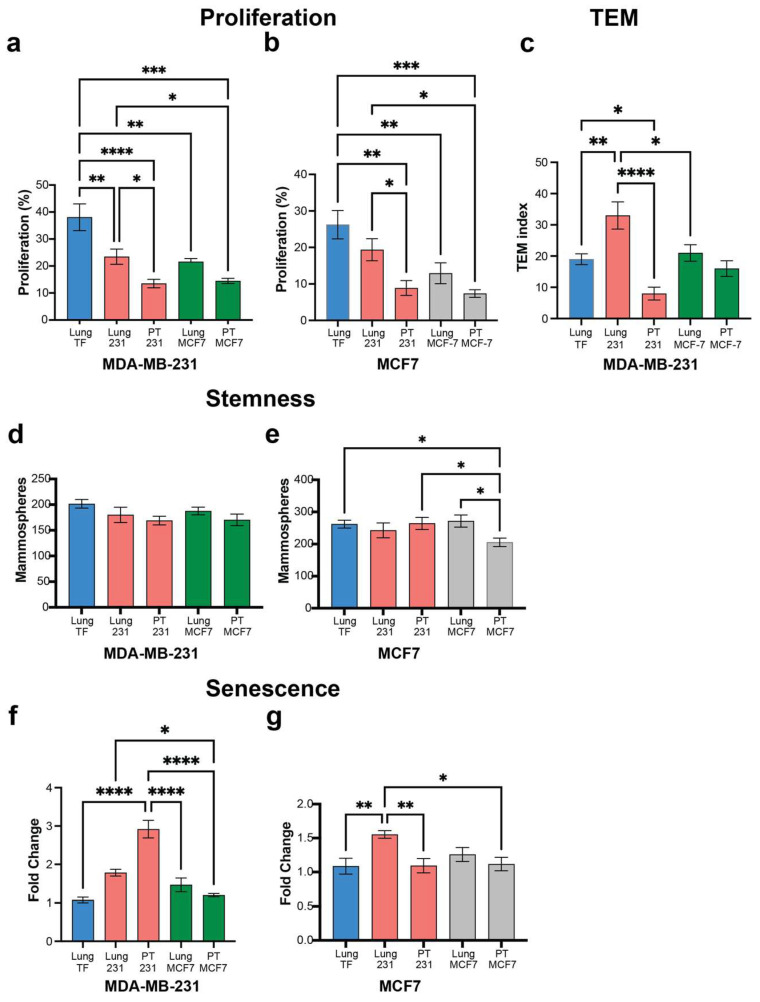
Non-metastatic tumors affect distant tissues promoting tumor cell growth, though not to the extent seen with highly metastatic tumors. Functional analysis of highly metastatic (MDA-MB-231) and non-metastatic (MCF7) tumor cells in contact with Conditioned Media (CM) from primary tumor or lungs from tumor-bearing or tumor-free mice (Control): (**a**) Cell proliferation assessment of MDA-MB-231 cells incubated with CM from MDA-MB-231- or MCF7-tumor-bearing tissues (PT and Lung). (**b**) Cell proliferation assessment of MCF7 cells incubated with CM from MDA-MB-231- or MCF7-tumor-bearing tissues. (**c**) Migration (TEM) assay of MDA-MB-231 cells incubated with CM from MDA-MB-231- or MCF7-tumor-bearing tissues. (**d**) Stemness analysis of MDA-MB-231 cells incubated with CM from MDA-MB-231- or MCF7-tumor-bearing tissues. (**e**) Stemness analysis of MCF7 cells incubated with CM from MDA-MB-231- or MCF7-tumor-bearing tissues. (**f**) Senescence assessment of MDA-MB-231 cells incubated with CM from MDA-MB-231- or MCF7-tumor-bearing tissues. (**g**) Senescence assessment of MCF7 cells incubated with CM from MDA-MB-231- or MCF7-tumor-bearing tissues. Comparison of two groups was performed with an unpaired, two-tailed Student *t*-test. Comparisons of three or more groups were analyzed by ANOVA with a Tukey posttest. * *p* < 0.05, ** *p* < 0.01, *** *p* < 0.001, **** *p* < 0.0001.

We next investigated non-metastatic tumors from the MCF-7 cell line for their impact on the distant environment. MCF-7 cells were inoculated, and CM was generated as described previously for inoculation with MDA-MB-231 cells. Ex vivo cultures were analyzed with CM from MCF-7 tumor-bearing mice applied to MCF-7 cells, with cultures using MDA-MB-231 cells used as a reference. CM from the primary tumor and distant tissues (i.e., lung) of the non-metastatic MCF-7 cells reduced the proliferation of MDA-MB-231 cells similar to the CM from the primary tumor and lung of MDA-MB-231-bearing mice, both of which had decreased proliferation relative to CM from tumor-free mice (Figure 6a). A similar trend was observed with the ex vivo culture of MCF-7 cells with the CM derived from MDA-MB-231-bearing mice. Cell proliferation was significantly reduced with CM from tumor-bearing mice relative to tumor-free animals with the lung and PT of MCF-7-bearing mice inducing similar proliferation as the MDA-MB-231-bearing mice (Figure 6b). Transendothelial migration was investigated using MDA-MB-231 cells, as the MCF-7 cells did not migrate in any condition tested. Notably, CM from the lungs of MDA-MB-231-bearing mice induced a substantially greater TEM activity response compared to CM from the lungs of tumor-free mice or lungs from mice bearing MCF7 cells (Figure 6c). The TEM levels for the CM from the lungs of MCF7-bearing mice were comparable to the TEM from tumor-free mice. High stemness activity was observed in both MDA-MB-231 and MCF7 cells across groups (Figure 6d,e). Stemness for MDA-MB-231 cells was consistent under tumor-free and tumor-bearing CM derived from MDA-MB-231- and MCF-7-inoculated mice (Figure 6d). Similarly, MCF7 cells presented a high and maintained a high stemness index across CM, with the exception of the microenvironment from PT of MCF7-inoculated mice, in which MCF7 had a minor but significant stemness activity decrease (Figure 6e). Finally, we evaluated the senescence of tumor cells incubated with tumor-free or tumor-bearing mice inoculated with MDA-MB-231 or MCF7 cells (Figure 6f,g). CM derived from MDA-MB-231 inoculated mice, promoted an increase in cell senescence of MDA-MB-231, with no significant change when incubated with MCF7 CM relative to healthy tissue (Figure 6f). Interestingly, the MCF7 cells had an increase in cell senescence only with lung CM from tumor-bearing mice inoculated with MDA-MB-231 cells (Figure 6g). Similar assays were executed with two additional non-metastatic cell lines, T47D and MDA-MB-134, and results were consistent with those obtained with the MCF7 cells (Supplementary Figure S1). These results demonstrate that non-metastatic tumors can impact the function of distant tissues, though the effects from these tumors are not of the same magnitude as the highly metastatic cells.

**Figure 7:**
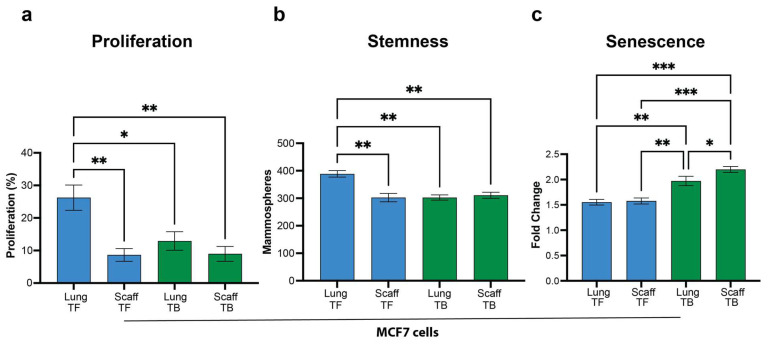
Scaffolds are conditioned by non-metastatic tumors similar to the lung. Functional analysis of healthy and tumor-induced natural and synthetic (lung and scaffold) microenvironment on MCF7 cells: (**a**) Cell proliferation assessment of MCF7 tumor cells incubated with healthy and tumor-induced microenvironment tissues (lung and scaffold). (**b**) Stemness and (**c**) Senescence. Comparison of two groups was performed with an unpaired, two-tailed Student *t*-test. Comparisons of three or more groups were analyzed by ANOVA with a Tukey posttest. * *p* < 0.05, ** *p* < 0.01, *** *p* < 0.001.

We next investigated whether non-metastatic primary tumors would condition the engineered tissue similarly to the endogenous tissue (lung). CM was derived from scaffolds implanted in mice that had been inoculated with MCF-7 cells, and cultured ex vivo with MCF-7 cells. CM from the synthetic niche of tumor-bearing mice significantly reduced tumor cell proliferation relative to synthetic niches from tumor-free mice. In addition, the cell responses to the CM from the scaffold were remarkably similar to what was observed with the conditioned media from the lung of MCF-7-tumor-bearing mice (Figure 7a). CM from MCF7-tumor-bearing mice significantly reduced the capacity to form spheroids relative to healthy lung microenvironment. However, CM generated from scaffolds of tumor-bearing mice was remarkably similar to CM from both the healthy scaffold and the tumor-bearing lung (Figure 7b). Senescence was comparable for both the synthetic and endogenous niche CM from MCF-7-inoculated mice (Figure 7c). These results confirm that the scaffolds are conditioned similarly to the lungs even by the non-metastatic MCF-7 cells. This result suggests that in the absence of metastases, cancer cells can still influence the microenvironment in distant tissues, producing cellular signals with the potential to modify the tumor cell phenotype.

### 3.5. Tumor-Induced Distant Tissue Microenvironments Lead to Pro-Tumor Cellular Signals by TC Transcriptional Activity Modulation That Are Recapitulated in a Synthetic Metastatic Niche

**Figure 8:**
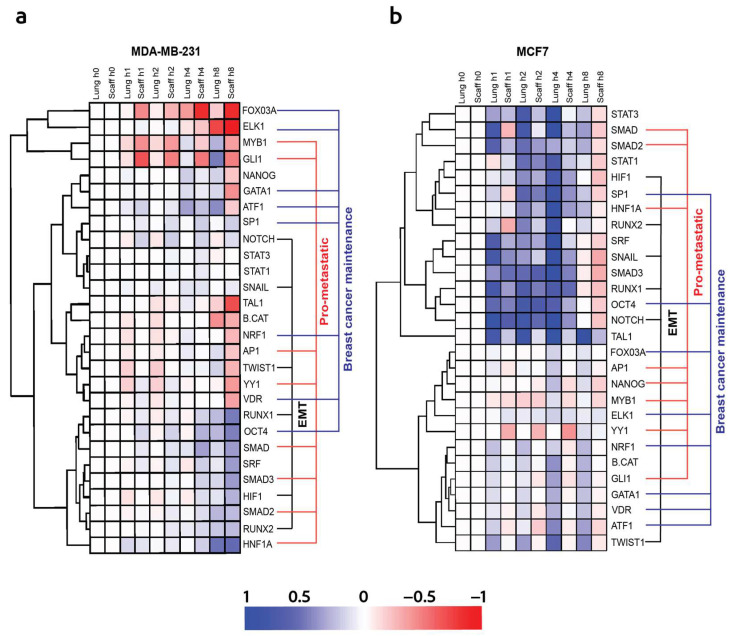
Tumor cells influence distant tissue microenvironments promoting a pro-tumor cellular signaling that can be recapitulated in a synthetic metastatic niche. Dynamic transcriptional activity of Transcriptional Factors (TrFs) associated with EMT (TWIST1, SNAIL1, RUNX1, RUNX2, HIF1, and NOTCH1), breast cancer maintenance (OCT4, SP1, GATA1, ATF1, FOXO3a, ELK1, VDR, and NRF1), pro-metastatic responses (SMAD2/3, HNF1a, GLI1, NANOG, YY1, MYB1, and AP1), and immune modulation (STAT1/3): (**a**) TrF activity induced by CM from lung and scaffolds from MDA-MB-231-tumor-bearing mice. (**b**) TrF activity induced by CM from lung and scaffolds from MCF7-tumor-bearing mice.

The similar trends in cell responses between the microenvironment signals from distant tissues and the scaffolds led to additional TRACER studies being conducted to investigate TrF activity beyond the EMT alone that was investigated earlier. MDA-MB-231 cells were infected with the TrF reporter and incubated with CM from MDA-MB-231- and MCF-7-tumor-bearing mice. We employed 32 TrF reporters known to be associated with a range of cellular processes critical to cancer progression. These include EMT (TWIST1, SNAIL1, RUNX1, RUNX2, HIF1, and NOTCH1), breast cancer maintenance (OCT4, SP1, GATA1, ATF1, FOXO3a, ELK1, VDR, and NRF1), pro-metastatic responses (SMAD2/3, HNF1a, GLI1, NANOG, YY1, MYB1, and AP1), and immune modulation (STAT1/3) (Figure 8). Transcription factor activity stimulated by the conditioned media from the scaffolds exhibited a trend similar to TrF activity induced by the lung tissues in tumor cells incubated with microenvironments from MDA-MB-231-tumor-bearing mice. A detailed analysis of the EMT-related transcription factors, including RUNX1, RUNX2, SNAIL, NOTCH1, and HIF1, revealed a gradual increase in their activity levels initiating at the 4 h time point and sustained until 8 h post-CM incubation. In parallel, the transcription factor activity related to pro-metastatic response (SMAD1,2,3 and HNF1a) and cancer cell maintenance (OCT4, and ATF1) was similarly increased at 4 h and remained pronounced through the 8 h time point following CM incubation. These findings suggest that the metastatic microenvironment induces the dynamic activity of an array of transcription factors related to cancer progression (Figure 8a). Conversely, the conditioned media derived from lungs and scaffolds of MCF7-tumor-bearing mice induced the rapid transcription factor activity of EMT (HIF1, RUNX1, RUNX2, NOTCH1, and SNAIL), pro-metastatic (SMAD1, SMAD2, and HNF1a), and breast cancer maintenance (SP1, and OCT4)-related factors, which were initiated minutes after CM incubation and were sustained until the 4 h timepoint (Figure 8b). TrF activity often occurs during early stages that influence subsequent cellular processes, including those leading to EMT and cancer-cell maintenance [25].

## 4. Discussion

Metastasis is a complex process mediated by intrinsic factors within tumor cells and extrinsic factors from the environment, which drive the dissemination and progression of the disease. Tumor-cell-intrinsic factors such as genomic landscape, mutation accumulation, chromosomal instability, and tumor heterogeneity play important roles in the progression, dissemination, and maintenance of the disease [26,27,28,29]. Nevertheless, the extrinsic environmental factors encompassed by immune cells, stromal cells, and their secreted molecules promote a cell signaling network that can influence metastasis formation and progression [30]. In this study, we investigated the microenvironment induced by the primary tumor in distant tissues and assessed the functional impact of the microenvironment on the cancer cell phenotype. The tumor microenvironment (TME) contributes to cancer development and progression through processes such as angiogenesis, immune escape, and metastasis, and thus contains information that could be useful in selecting therapeutic strategies [31]. Herein, we analyzed the functional characteristics of the microenvironment that is conditioned by metastatic and non-metastatic tumors and compared these functional characteristics between endogenous and engineered tissue. In this study, NSG mice were selected for inoculation with the human cells to support their engraftment without rejection. NSG mice have innate immune cells yet lack adaptive immune cells (B, T, and NK cells) [32], with the latter supporting the transplantation of human cells without rejection. The innate immune cells initiate an inflammatory response to the implant and contribute to inflammation associated with cancer progression, though these responses do not include signaling from adaptive immune cells. We have previously reported the immune cell composition in the lungs and scaffolds for two immune-competent models of triple-negative breast cancer. The 4T1 and E0771 models exhibit a significant upregulation of neutrophils, which is consistent with the results obtained with the 231 model. While the absence of adaptive cells in the NSG mice does not support the cross-talk between T cells and neutrophils, the general trend of increased abundance of neutrophils is maintained [33]. Additionally, we have previously demonstrated that the increased migration of 231 or 4T1 tumor cells is similar for conditioned media from tumor-bearing NSG mice or tumor-bearing BALB/c, respectively [15]. Building upon this foundation, the current study focused on the primary tumor conditioning of distant tissues, and their impact on the number of innate immune cells and their impact on the microenvironment.

We demonstrated that both metastatic and non-metastatic primary tumors alter the properties of distant tissues, which can activate cellular processes that support cancer progression, though the extent of activation is distinct between the metastatic and non-metastatic tumors. The alterations to the microenvironment in distant tissues can stimulate a pro-invasive tumor cell phenotype by the activation of transcription factors related to the EMT process to a different extent. Recent reports suggest that the EMT process, widely observed in cancer progression, is not a binary switch but rather a spectrum of transitional states, each consisting of unique characteristics and contributing differently to the metastatic potential and disease progression. Tumor cells that express a mix of the EMT phenotype are found to be more efficient at migration, colonizing secondary sites, and promoting metastasis [8]. Tumors from either MDA-MB-231 or MCF7 modify the environment in distant tissues. Intrinsically, the metastatic cell lines are distinct from the non-metastatic cell lines in many aspects. MDA-MB-231 cells have functional mutations in senescence genes that prevent the tumor cell from entering a final senescence state [22], whereas MCF7 cells are considered a homogeneous cell line without the capacity for migration and invasion [34]. Nevertheless, both high-metastatic and non-metastatic cell lines promoted the conditioning of distant tissues whose dynamics induced signals that led to the persistent activation of TrFs that are associated with EMT, consistent with a more invasive and migratory phenotype in the tumor cells. The microenvironment derived from the non-metastatic MCF7 cell line is capable of inducing the rapid TrF activity of factors related to the invasiveness and aggressiveness of TCs. However, the conditions generated from the high metastatic MDA-MB-231 cells were gradual but maintained TrF activity induction that translated into a more aggressive and migratory TC phenotype.

We compared the biology of the endogenous metastatic niche with the biology within an engineered tissue that offers a potential surrogate to endogenous metastatic sites. The current clinical assessment of the TME and its impact on tumor cells requires taking samples of the identified metastatic site, which is associated with significant risk to the patient’s vital organs such as the lung. Liquid biopsy is being investigated for its ability to provide information about cancer progression, which is often limited to late-stage disease to obtain sufficient cfDNA or circulating tumor cells (CTCs) for analysis. Additionally, the tumor cells are separated from the microenvironment and thus liquid biopsy does not provide information about the microenvironment. We have previously reported on an implantable scaffold that can function as a metastatic niche containing both immune cells and tumor cells and can be sampled from the subcutaneous space with minimal risk [14]. This engineered tissue becomes vascularized and can recruit large numbers of immune cells, and the initial recruitment of innate immune cells leads to signaling that can influence tumor cell recruitment and other responses. Using real-time TrF activity measurements, we monitored the activation of several key factors from the microenvironment that play a role in cellular processes like cancer cell maintenance and EMT. Importantly, the signaling assessed from the cells in the scaffold was consistent with the signaling induced by the endogenous metastatic site. Furthermore, the scaffold microenvironment from the non-metastatic cell line MCF7 showed a similar TrF activity pattern from the corresponding lung microenvironment, indicating that the implant can function as a surrogate microenvironment for both metastatic and non-metastatic cells. The ability to have an engineered tissue in the subcutaneous space has the potential to substantially reduce the risk of biopsy and our results herein suggest that our engineered implants successfully recapitulate key aspects of the tumor cells and microenvironment dynamics.

The development of metastases is a highly dynamic process that initiates with the conditioning of distant tissue by the primary tumor, which leads to the recruitment of immune cells that prepare the site for subsequent colonization by tumor cells [35]. The metastatic sites may initially be metastatically dormant, which is characterized as having a stable number of tumor cells, yet may transition to a metastatic progression that is characterized by tumor cell expansion in clonal or polyclonal clusters [36]. The engineered tissue may enable longitudinal monitoring of the metastatic microenvironment to assess the dynamics of disease progression [37]. Herein, analysis of the immune cells in the scaffold indicates that the immune cell types present in the scaffold are similar to the immune cell types found within the lung, indicating that the scaffolds and lungs are similarly conditioned by metastatic and non-metastatic tumors. Furthermore, the endogenous and engineered niche provide similar responses to the tumor cells despite differences in the stromal cell population of each (e.g., fibroblasts, macrophages), suggesting that the circulating immune cells recruited to these sites are major drivers of the TME [38].

## 5. Conclusions

The study of the dynamics of the microenvironment within tumor-induced distant tissues indicates that fundamental information about the stage and aggressiveness of cancer may be identified. In addition to immune cells, the scaffold captures tumor cells that have been shown to be similar to disseminated tumor cells or tumor cells in the lung, which are distinct from circulating tumor cells with low metastatic potential [8,39]. Herein, we indicate that the immune cells, which have a significant role in tumor progression, can be isolated and readily analyzed using standard techniques. The immune cells from metastatic and non-metastatic tumors have some similarities regarding cell composition and signaling, yet do not stimulate cancer progression to the same extent. The engineered tissue provides a resource to obtain cells reflective of the endogenous metastatic site without significant risk to the patient, and the analysis of these cells may provide an opportunity to detect disease or guide the management of patients.

## Data Availability

All data are available in the main text or the Supplementary Materials. Source data, including data for TRACER, are provided with this paper.

## Supplementary Materials

The following supporting information can be downloaded at: https://www.mdpi.com/article/10.3390/cancers16040796/s1, Figure S1: Non-metastatic cell lines T47D and MDA-MB-134 affect distant tissues promoting tumor cell growth; however, they do not induce transendothelial migration to the extent as high-metastatic cell line. Functional analysis of highly metastatic (MDA-MB-231) and non-metastatic (T47D and MDA-MB-134) tumor cell lines in contact with respective CM from primary tumor or lungs from tumor-bearing or tumor-free mice (Control): (a) Cell proliferation assessment of MDA-MB-231 cells incubated with CM from MDA-MB-231- or T47D-tumor-bearing tissues (PT and Lung). (b) Cell proliferation assessment of MDA-MB-231 cells incubated with CM from MDA-MB-231- or MDA-MB-134-tumor-bearing tissues (PT and Lung) (c) Transendothelial Migration (TEM) assay of MDA-MB-231 cells incubated with CM from MDA-MB-231- or T47D-tumor-bearing tissues. (d) Transendothelial Migration (TEM) assay of MDA-MB-231 cells incubated with CM from MDA-MB-231- or MDA-MB-134-tumor-bearing tissues. Comparison of two groups was performed with an unpaired, two-tailed Student *t*-test. Comparisons of three or more groups were analyzed by ANOVA with a Tukey posttest. A *p*-value < 0.05 was considered significant. Figure S2: Schematic of cell flow cytometry gating strategy used to characterize the innate immune cell response: (a) Gating strategy for macrophages: CD11b+CD64+F4/80+SiglecF- and dendritic cells: CD64-CD11b+CD11c+MHCII+. (b) Gating strategy for monocytes: CD45+CD11b+SiglecF- Ly6Chigh. (c) Gating strategy for neutrophils: SSChigh CD11b+ SiglecF- GR-1+. Figure S3: Representative pictures of H&E staining of (a,b) Lung and (b,c) Scaffold from tumor-free and tumor-bearing mice at 4X and 20X magnification.
