# Supplementary material for: Human Breast Cancer Cell Lines Differentially Modulate Signaling from Distant Microenvironments, Which Reflects Their Metastatic Potential
Source: Cancers (Basel). 2024 Feb 15;16(4):796. doi: 10.3390/cancers16040796 (PMC10887178; doi:10.3390/cancers16040796)
Supplement: Supplementary file 1 [file cancers-16-00796-s001.zip › cancers-2759351-supplementary.pdf]

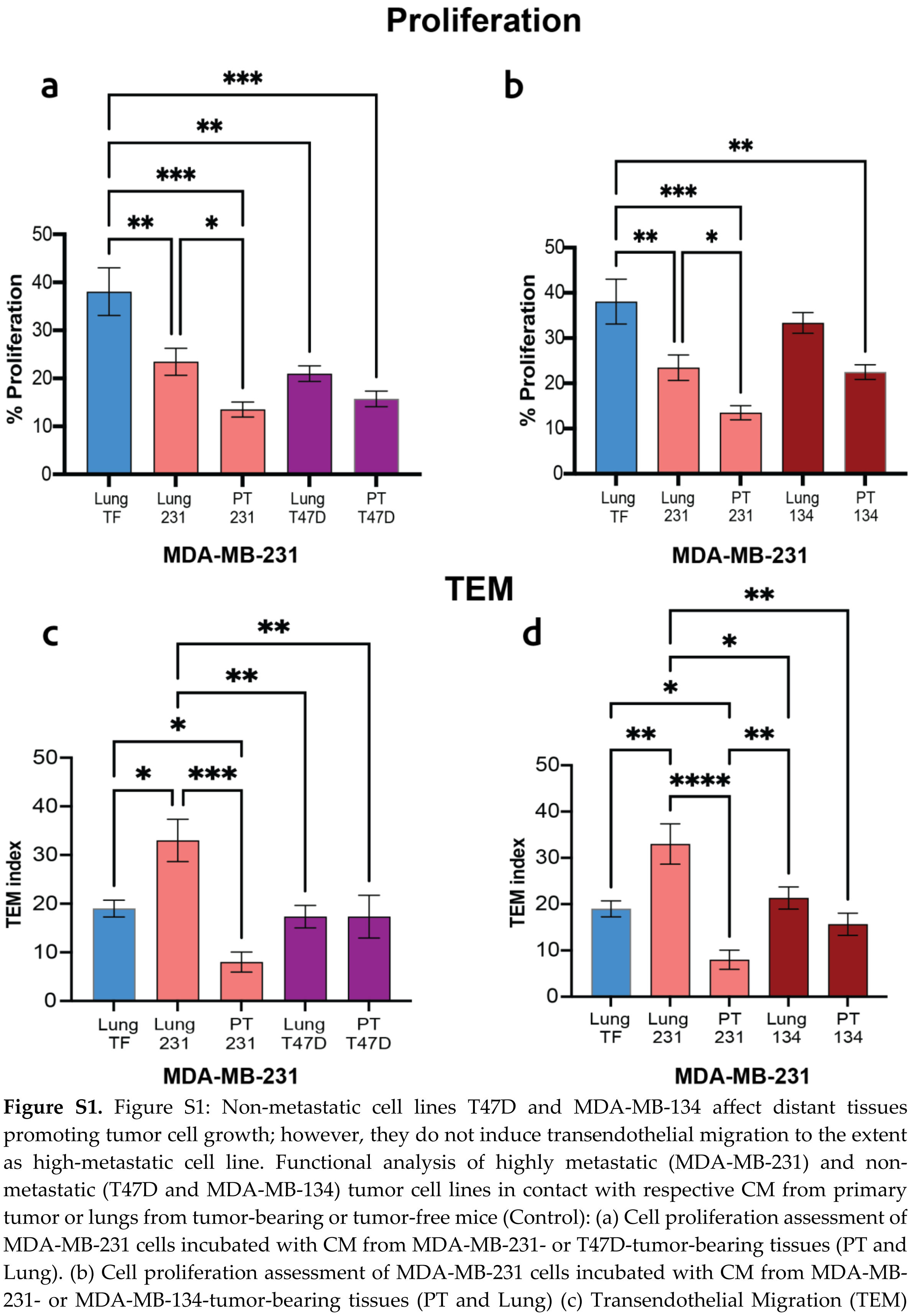

Figure S1. Figure S1: Non-metastatic cell lines T47D and MDA-MB-134 affect distant tissues promoting tumor cell growth; however, they do not induce transendothelial migration to the extent as high-metastatic cell line. Functional analysis of highly metastatic (MDA-MB-231) and non-metastatic (T47D and MDA-MB-134) tumor cell lines in contact with respective CM from primary tumor or lungs from tumor-bearing or tumor-free mice (Control): (a) Cell proliferation assessment of MDA-MB-231 cells incubated with CM from MDA-MB-231- or T47D-tumor-bearing tissues (PT and Lung). (b) Cell proliferation assessment of MDA-MB-231 cells incubated with CM from MDA-MB-231- or MDA-MB-134-tumor-bearing tissues (PT and Lung) (c) Transendothelial Migration (TEM) assay of MDA-MB-231 cells incubated with CM from MDA-MB-231- or T47D-tumor-bearing tissues. (d) Transendothelial Migration (TEM) assay of MDA-MB-231 cells incubated with CM from MDA-MB-231- or MDA-MB-134-tumor-bearing tissues. Comparison of two groups was performed with an

unpaired, two-tailed Student *t*-test. Comparisons of three or more groups were analyzed by ANOVA with a Tukey posttest. A *p*-value < 0.05 was considered significant.

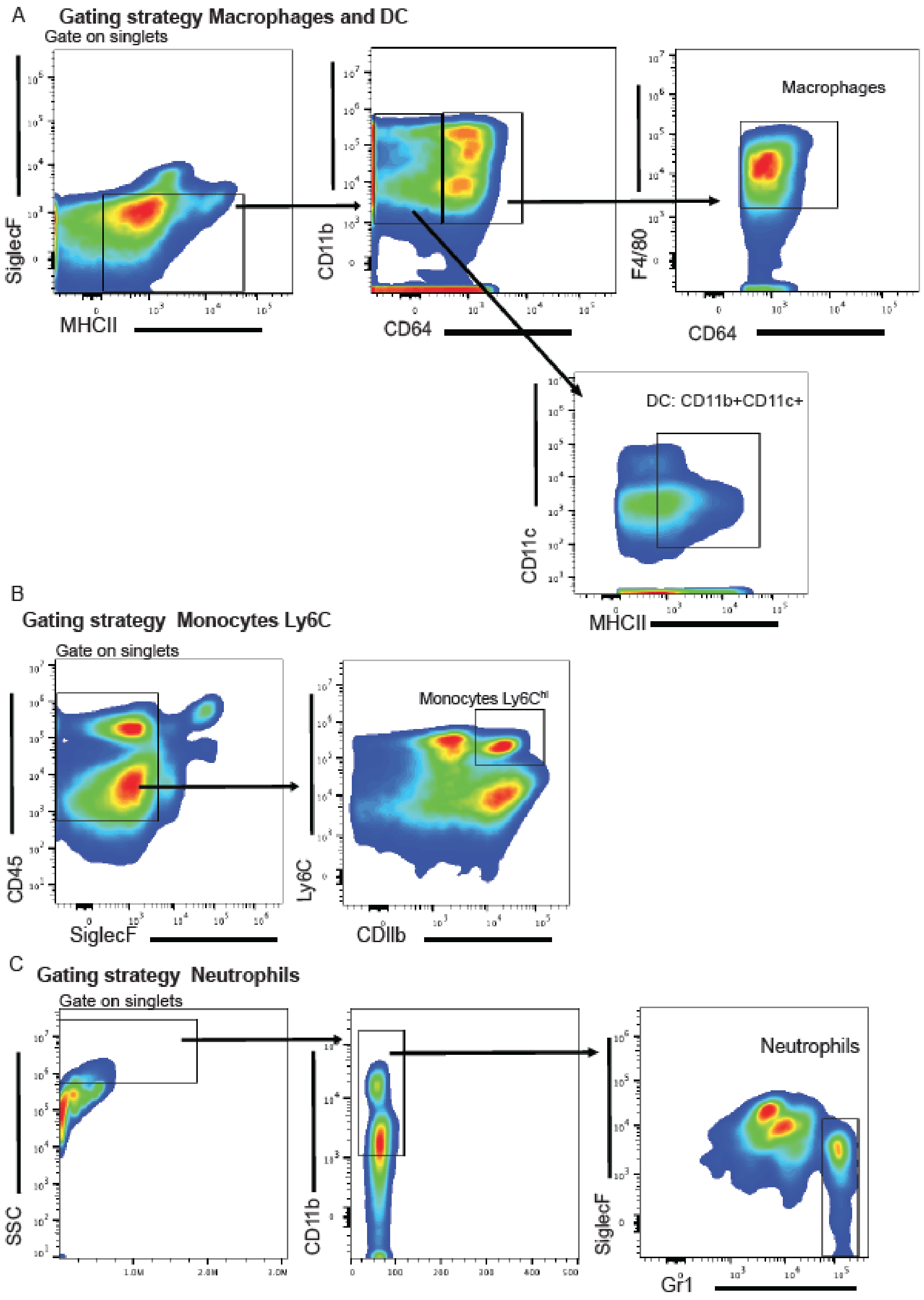

Figure S2. Schematic of cell flow cytometry gating strategy used to characterize the innate immune cell response: (a) Gating strategy for macrophages: CD11b+CD64+F4/80+SiglecF- and dendritic cells: CD64-CD11b+CD11c+MHCII+. (b) Gating strategy for monocytes: CD45+CD11b+SiglecF- Ly6Chigh. (c) Gating strategy for neutrophils: SSChigh CD11b+ SiglecF- GR-1+.

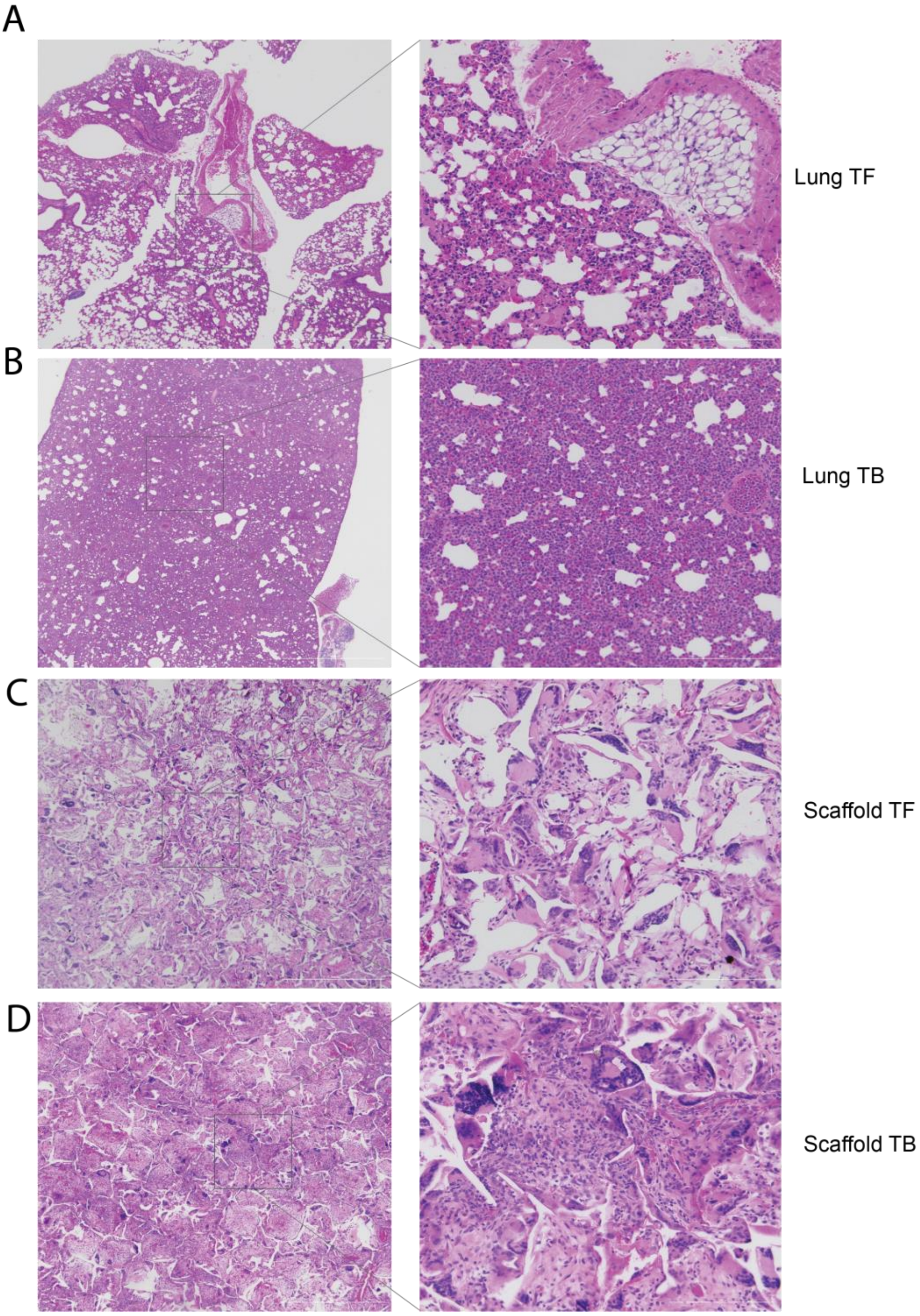

Figure S3. Representative pictures of H&E staining of (a,b) Lung and (b,c) Scaffold from tumor-free and tumor-bearing mice at 4X and 20X magnification.
